# First Report from the Asian Rotavirus Surveillance Network

**DOI:** 10.3201/eid1006.030519

**Published:** 2004-06

**Authors:** Joseph Bresee, Zhao-Yin Fang, Bei Wang, E.A.S. Nelson, John Tam, Yati Soenarto, Siswanto Agus Wilopo, Paul Kilgore, Jung Soo Kim, Jung Oak Kang, Wong Swee Lan, Chan Lee Gaik, Kyaw Moe, Kow-Tong Chen, Chuleeporn Jiraphongsa, Yaowapa Pongsuwanna, Nguyen Van Man, Phan Van Tu, Le Thi Luan, Erik Hummelman, Jon R. Gentsch, Roger Glass, the members of the Asian Rotavirus Surveillance Network

**Affiliations:** * Centers for Disease Control and Prevention, Atlanta, Georgia, USA;; † Chinese Center for Disease Control and Prevention, Beijing, People's Republic of China (PRC);; ‡ Southeast University, Nanjing, PRC;; § Chinese University of Hong Kong, Hong Kong, Special Administrative Region (SAR), PRC;; ¶ Gadjah Mada University, Yogyakarta, Indonesia;; # International Vaccine Institute, Seoul, Korea;; ** Chonbuk National University Medical School, Chonju, Korea;; †† Hanyang University School of Medicine, Seoul, Korea;; ‡‡ Institute of Pediatrics, Kuala Lumpur, Malaysia;; §§ Kuching Hospital, Kuching, Malaysia;; ¶¶ Ministry of Health, Yangon, Myanmar;; ## Department of Health, Taipei, Taiwan;; *** Ministry of Public Health, Nonthaburi, Thailand;; ††† Poliomyelitis Vaccine Research and Production Center, Hanoi, Vietnam;; ‡‡‡ Pasteur Institute, Ho Chi Minh City, Vietnam

**Keywords:** rotavirus, rotavirus vaccines, diarrhea, Asia, surveillance

## Abstract

Rotavirus remains the most common cause of severe, dehydrating diarrhea among children worldwide. Several rotavirus vaccines are under development. Decisions about new vaccine introduction will require reliable data on disease impact. The Asian Rotavirus Surveillance Network, begun in 2000 to facilitate collection of these data, is a regional collaboration of 36 hospitals in nine countries or areas that conduct surveillance for rotavirus hospitalizations using a uniform World Health Organization protocol. We summarize the Network's organization and experience from August 2001 through July 2002. During this period, 45% of acute diarrheal hospitalizations among children 0–5 years were attributable to rotavirus, higher than previous estimates. Rotavirus was detected in all sites year-round. This network is a novel, regional approach to surveillance for vaccine-preventable diseases. Such a network should provide increased visibility and advocacy, enable more efficient data collection, facilitate training, and serve as the paradigm for rotavirus surveillance activities in other regions.

In recent years, several international agencies, including the World Health Organization (WHO), the Global Alliance for Vaccines and Immunization (GAVI), and the Children's Vaccine Program at the Program for Appropriate Technology in Health (PATH), have identified the accelerated development and introduction of a rotavirus vaccine to be among their highest priorities. This decision was made based on the high incidence of rotavirus, the most common cause of severe diarrhea in children worldwide. An estimated 440,000 children die of rotavirus each year (*1*), and in developing countries, 5% of all deaths in children <5 years of age are due to rotavirus. Furthermore, rotavirus is responsible for 25% to 50% of all hospitalizations of children for diarrhea in both industrialized and developing countries (*2*). After 2 decades of vaccine development and testing, the principles for making safe and effective, live oral vaccines have been firmly established, and several new candidate vaccines are currently in the late stage of development (*3*). Given the importance of rotavirus, GAVI has initiated the Accelerated Development and Introduction Program to expedite the development, evaluation, and introduction of rotavirus vaccines into the poorest countries with the goal of preventing most rotavirus deaths and hospitalizations within the next decade.

Despite the global awareness about the prevalence of rotavirus, physicians and policymakers in most developing countries, where rotavirus causes the most fatalities and cases of severe disease and where new vaccines could have their greatest value, know little about rotavirus in their location. While these physicians and policymakers may appreciate that diarrhea is the first or second leading cause of death in children <5 years of age, the diagnosis of rotavirus is rarely, if ever, made at the local level. Moreover, acute diarrhea is treated with rehydration, regardless of the cause, so a diagnosis is not required, and no specific means of preventing rotavirus currently exists. Consequently, rotavirus is not often viewed as a priority and is incorrectly considered a disease that can be prevented by improvements in water and sanitation. If a vaccination program against rotavirus is to be successful, local leaders must understand the disease, which might include initiating surveillance of rotavirus, assessing the full incidence of rotavirus disease, and appreciating the impact that a new vaccine might have if added to the existing program of childhood immunizations.

In an effort to create regional awareness of the incidence of rotavirus disease and let countries assess the potential value of rotavirus vaccines currently being developed, we established the Asian Rotavirus Surveillance Network (ARSN), the first regional collaboration of its kind, as a pilot program for epidemiologic surveillance and advocacy. As members of this network, participating countries in the region conduct sentinel hospital surveillance to monitor rates of rotavirus among children hospitalized for diarrhea in several major hospitals by using a common protocol and comparable diagnostic test. The goal is to understand the epidemiology and impact of rotavirus diarrhea in the region, to educate physicians and health leaders about the importance of this problem, and let them assess the potential public health value of introducing vaccines. The same surveillance system could ultimately be used to monitor the impact of a vaccination effort.

Although many of the participating sites had begun surveillance up to 6 months earlier, we report preliminary data for the first year of the network, in which all but one country had completed a full year of surveillance, August 2001–July 2002. Data from Korea are not included because investigators in that country began surveillance in June 2002 and did not have a full year's data to report. We report data from > 16,000 children hospitalized for diarrhea and tested for rotavirus in 33 hospitals in eight Asian countries or regions. Our results document the rates of rotavirus in the region, identify features in the epidemiology of the disease that might effect future immunization efforts, and underscore the value of regional surveillance networks to collect data with the same protocol and to test specimens with comparable diagnostic assays.

## ARSN

ARSN is a collaboration of investigators from medical centers and public health agencies in nine Asian countries or regions. The goal of the group is to define the epidemiology and rates of rotavirus disease in Asia and to use these data to make informed decisions regarding the possible future use of rotavirus vaccines. ARSN was formed in 2000 in response to a WHO report that called for expedited rotavirus vaccine evaluation and introduction in Asia (*4*). WHO commissioned a generic protocol for sentinel hospital surveillance of rotavirus that would allow investigators in many countries to assess, in a simple, economical, and timely fashion, the epidemiology and disease prevalence of rotavirus (*5*). This protocol provides the minimum requirements for hospital-based surveillance, with attention to collecting and testing fecal samples, and includes an appendix to identify the catchment population for the hospital, suggestions to assess the prevalence of fatal rotavirus disease, and a discussion of the methods to characterize rotavirus strains.

## Goals and Organizational Structure

The goals of ARSN are to use the generic protocol as a basis to: 1) define the epidemiology and strain distribution of rotavirus in participating countries, 2) estimate the costs associated with rotavirus and its prevalence in these settings, 3) create a surveillance system that can monitor the effectiveness of a vaccine program once introduced, 4) serve as a basis to conduct enhanced surveillance with special studies (e.g., cost-benefit or death rate studies) to inform decision makers considering a new vaccine program, and 5) describe trends in rotavirus activity and strain distribution over a large geographic region over time.

ARSN consists of 13 collaborating institutions in nine countries or regions that conduct hospital-based surveillance to monitor the rate of rotavirus infection among children hospitalized with diarrhea. The network is supported by five international donor groups, and coordination is provided by the Centers for Disease Control and Prevention (CDC) (Table 1). The lead groups in each country are responsible for establishing a hospital surveillance network, ensuring the timely collection of data, creating the laboratory capacity to test fecal specimens for rotavirus and to characterize strains identified, performing the analysis, and writing the report. A summary of all surveillance data is submitted electronically to CDC each month, and CDC prepares and distributes a quarterly report to all participants.

**Table 1:** Member institutions and participating hospitals of the Asian Rotavirus Surveillance Network

Sites	Lead institution(s)	Participating hospitals
China	Institute of Virology, Chinese Center for Disease Control, Ministry of Health, Beijing Southeast University, Nanjing International Vaccine Institute	Beijing Friendship Hospital Changchun Children's Hospital Lulong County Hospital Lulong Maternal and Child Health Hospital Kunming Hospital Ma-An-Shan Steel Trust Hospital Suzhou University-Affiliated Children's Hospital
Hong Kong	Chinese University of Hong Kong, New Territories	Prince of Wales Hospital Queen Elizabeth Hospital Tuen Mun Hospital Pamela Youde Nethersole Eastern Hospital
Indonesia	Gadjah Mada University, Yogyakarta	Dr. Sardjito Teaching Hospital, Yogyakarta Wirosaban District Hospital, Yogyakarta Purworejo Hospital, Purworejo
Korea	Chonbuk National University International Vaccine Institute, Seoul	ChungEub Asan Foundation Medical Center, Chonbuk National University Hospital Chonju Presbyterian Medical Center
Malaysia	Institute of Pediatrics, Kuala Lumpur University, Kuala Lumpur	Kuala Lumpur Hospital, Kuala Lumpur Kuching Hospital, Kuching, Sarawak
Myanmar	Department of Medical Research, Ministry of Health	Yangon Children's Hospital, Yangon
Taiwan	Taiwan Center for Disease Control, Taipei	National Taiwan University Hospital, Taipei Veteran General Hospital, Taipei Veteran General Hospital, Taichung Veteran General Hospital, Kaohsiung
Thailand	Ministry of Public Health, Bangkok	Nongkhai Hospital Maesod Hospital Prapokklao Hospital Ramathibodi Hospital Hadyai Hospital Srakaew Hospital
Vietnam	National Institute of Hygiene and Epidemiology, Hanoi POLIOVAC, Hanoi Pasteur Institute, Ho Chi Minh City	St. Paul's Hospital, Hanoi Swedish Children's Hospital, Hanoi Children's Hospital, Hai Phong General Hospital, Khan Hoa General Pediatric Hospital #1, Ho Chi Minh City General Pediatric Hospital #2, Ho Chi Minh City

The basic structure for surveillance of children hospitalized for diarrhea is described in the Generic Protocol available from the WHO website (*5*). In brief, it provides that all children <59 months of age admitted to a participating hospital with physician-diagnosed acute diarrhea of <7 days' duration be surveyed. At admission, simple uniform data are collected from the medical records by using a standard questionnaire that includes date of admission, age and sex of the patient, symptoms of illness, and outcome. A fresh fecal specimen is obtained from each child, placed in a clean container, and stored at 4°C until tested for rotavirus with an enzyme immunoassay (EIA), either Rotaclone (Meridian Diagnostics, Inc., Cincinnati, OH) or DAKOPATTS (DAKO Diagnostics Ltd., Glostrup, Denmark), or by polyacrylamide gel electrophoresis (PAGE). Testing is conducted at the participating hospital laboratory or at the coordinating institution in the country. Each site tracks the proportion of total diarrheal admissions tested for rotavirus as an indicator of the sensitivity of the system to monitor disease. A subset of samples found to be positive for rotavirus is tested for G and P types by using reverse transcriptase–polymerase chain reaction (RT-PCR) according to published methods (*6*). Samples are selected for strain characterization on the basis of representativeness in terms of seasonal and age distribution and their quality. The generic protocol was pilot tested in Vietnam, and the encouraging results of this survey led to some simplifications and improvements in the study methods (*7*). Individual country proposals were reviewed and approved by ethical review boards in member institutions.

## Progress during the First Year

Surveillance began in Hong Kong in December 2000, and by August 2002, 36 hospitals in 26 cities or towns in nine Asian countries or regions were actively participating and routinely reporting their results (Table 1). Collaborators from Korea joined the network, with 3 hospitals reporting in June 2002. Of the 36 hospitals conducting surveillance, 19 (53%) are in large urban settings, 14 (39%) are in smaller cities, and 3 (8%) are rural hospitals. Seventeen (47%) are tertiary-care hospitals.

**Figure 1:**
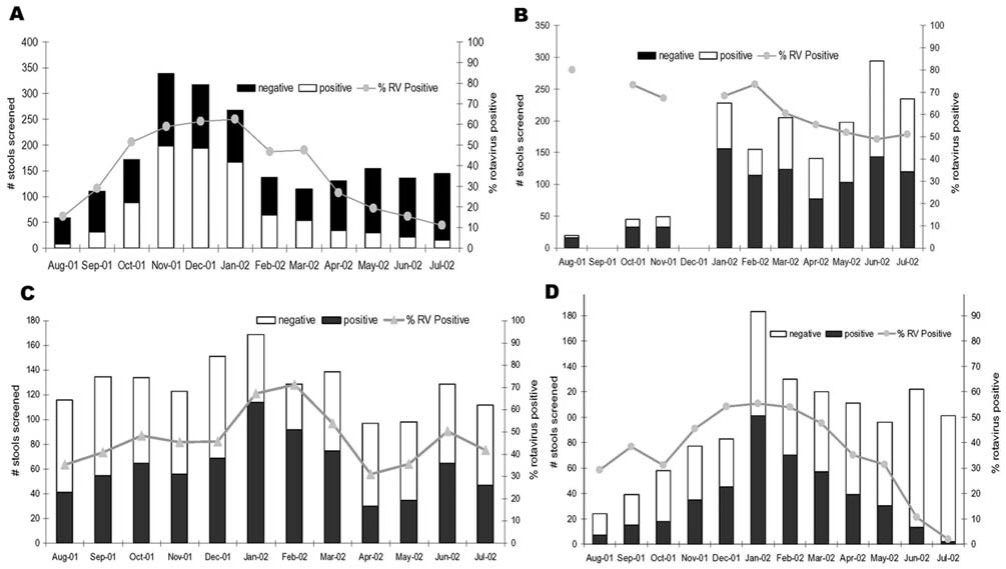
Seasonality of rotavirus (RV) in member countries of the Asian Rotavirus Surveillance Network. A, China; B, Vietnam; C, Taiwan; D, Thailand.

**Figure 2:**
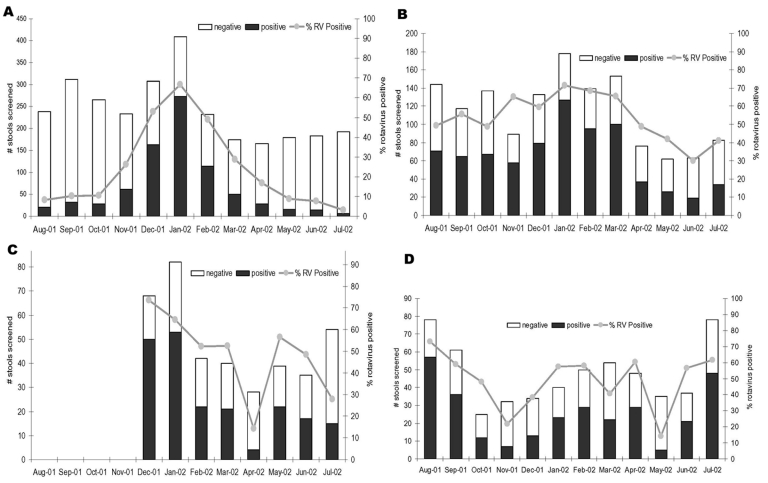
Seasonality of rotavirus (RV) in member countries of the Asian Rotavirus Surveillance Network. A, Hong Kong; B, Malaysia; C, Myanmar; D, Indonesia.

As of July 2002, seven of nine regions had collected 1 full year of data, and two collaborating countries (Korea and Myanmar) had collected data for <1 full year. From August 2001 through July 2002, a total of 11,498 stool samples were obtained from 16,173 patients, which represented more than 71% of all children admitted to participating hospitals with acute gastroenteritis (Table 2). Each month, participating hospitals collected fecal specimens from 679 to 1,556 children <5 years of age. Overall, rotavirus was detected in 45% (n = 5,124) of stools tested, with a range among hospitals of 18% to 67%. When only the sites that contributed 12 full months of data during this period were surveyed (to account for seasonal peaks of rotavirus disease), rotavirus was still detected in 44% of children tested. Rotavirus was detected year-round in all sites; however, wintertime peaks (November to March) were evident in the most northern areas (China, Taiwan, Hong Kong), while seasonal peaks were not as clearly defined in more southern sites, with tropical climates (Vietnam, Thailand, Malaysia) (Figure 1 and 2).

**Table 2:** Rates of rotavirus detection in Asian Rotavirus Surveillance Network sites, August 2001–July 2002

Sites	Start of surveillance	No. stool samples tested	% (no.) one rotavirus-positive sample	Range of % rotavirus-positive samples among participating hospitals
China	Aug 2001	2,079	44 (910)	24–65
Korea^a^	Jun 2002	N/A	N/A	N/A
Taiwan	Apr 2001	1,532	49 (744)	43–53
Hong Kong	Dec 2000	2,986	28 (829)	18–35
Vietnam	Feb 2001	1,570	59 (921)	47–67
Myanmar^b^	Dec 2001	388	53 (204)	53^c^
Thailand	Feb 2001	992	44 (436)	38–49
Malaysia	Feb 2001	1,374	57 (778)	52–59
Indonesia	Aug 2001	577	52 (302)	47–57
Overall		11,498	45 (5,124)	18–67

^a^Data from Korea absent due to start of surveillance in June 2002. ^b^Partial year of data. ^c^Single hospital in Myanmar participating in surveillance.

By mid-2002, five sites had begun strain characterization: China, Malaysia, Vietnam, Hong Kong, and Taiwan. CDC sponsored ARSN investigators from Indonesia, Taiwan, Vietnam, and Korea to train in Atlanta, and as a result, these sites have initiated genotyping of specimens from surveillance in these countries as of mid-2003. Finally, arrangements have been made for training a scientist from Myanmar in Hong Kong and will begin soon. CDC provided technical expertise, reagents, and protocols for some of the laboratory work and will provide assistance in resolving nontypeable strains.

## Discussion

ARSN was organized to allow for the timely and economic collection of quality data on the effect of rotavirus infections in Asia, to collect these data efficiently, and to facilitate use of the data to inform decision makers about possibly introducing rotavirus vaccines. During the first year of the collaboration, ARSN produced valuable data that also yielded some surprises, and it has contributed to local and regional training, capacity building, advocacy, and development of the infrastructure for surveillance in member sites. Preliminary data from ARSN reaffirm that rotavirus remains a major cause of severe gastroenteritis among infants and young children. Moreover, rotavirus predominates in all sites, whether urban or rural, north or south, industrialized or developing, regardless of a country's gross national product. In addition, unlike rotavirus hospitalizations in the United States (*8**,**9*) and Europe (*10*), those in the ARSN settings occurred year-round, although seasonal peaks in disease were observed in the northernmost sites, areas with more temperate climates. These findings are similar to those found in the summary from the African data of Cunliffe et al. (*2*). The finding that rotavirus disease occurs with high frequency in settings with a variety of sanitation conditions reinforces the hypothesis that vaccines, not improved hygiene and water quality, are the best strategy to prevent this disease (*3*).

The preliminary finding of such high rates of rotavirus in sites throughout the region among children hospitalized for diarrhea confirms pilot studies in Vietnam that identified rotavirus in >50% of patients hospitalized with diarrhea (*7*). These high rates have implications for our global estimates of the prevalence of rotavirus disease. The most recent estimates of the global prevalence of rotavirus disease were determined on the basis of a literature review of studies conducted in the late 1980s and 1990s that estimated the percentage of rotavirus detected among children hospitalized for diarrhea (*1*). In this study, the authors estimated that from 20% (for low-income countries) to 34% (for high-income countries) of hospitalizations for children >5 years were due to rotavirus infection. Data collected by the ARSN sites indicate higher rates of rotavirus illness in hospitalized children than used in previous models that have estimated rotavirus-associated disease and death globally. Indeed, the findings of the ARSN sites presented here are generally higher than those in previous studies in ARSN member countries that used similar methods (Table 3), but agree with recent data from studies in South America and Africa (*31**,**32*), and to recent rates from other investigators in Asia (*33*). One hypothesis for this difference is that improvements in sanitation and hygiene have reduced the number of diarrheal cases caused by bacteria and parasites, but less so the number due to rotavirus, because of differences in modes of transmission. As a result, the proportional fraction of diarrheal disease due to rotavirus rather than other causes increases as populations gain better access to clean water and sanitation. Among ARSN sites, rates of rotavirus detection in industrialized Hong Kong have changed little over the past decade, while countries with developing economies generally have found estimates higher than those from studies conducted in the 1970s and 1980s. Reports of bacterial enteric disease surveillance from some relatively high-income countries have demonstrated decreasing rates of disease (*34**–**36*); however, few data document trends of bacterial and parasitic enteric infections in industrialized countries. In addition, indirect support of the hypothesis comes from diarrheal death rates in Mexico during the 1990s (*37*) and the United States in the 1980s (*38*). In both settings, summer and winter peaks of diarrheal death rates have, over time, been replaced by single wintertime peaks. In Mexico, the decrease in summertime diarrheal death rates was associated with improvements in water supply. In both countries, wintertime rotavirus peaks in deaths have declined steadily over time but remain comparatively high.

**Table 3:** Comparison of results of rotavirus detection in hospitalized children from current and past studies

Site	Past studies			ARSN results	Difference
	Ref.	Y of study	% rotavirus positive	% rotavirus positive	% increase (decrease)
Taiwan	(*11*)	1996	27	49	22
	(*12*)	1991	43		6
	(*13*)	1984	15		34
	(*14*)	2002	41		8
China	(*15*)	1996–1999	26	44	18
	(*16*)	1995	41		3
	(*17*)	1983–1984	13		31
Malaysia	(*18*)	1988–1989	28	57	29
Thailand	(*19*)	1977–1996	30	44	14
	(*20*)	1983–1984	17		27
	(*21*)	1984–1985	55		(11)
	(*22*)	1985–1986	33		11
	(*22*)	1986–1987	25		19
	(*23*)	1987–1988	20		24
	(*24*)	1995–1996	17		26
	Pongsuwanna	1991–1994	38		6
Indonesia	(*25*)	1978–1979	38	53	15
Myanmar	(*17*)	1982–1983	22	53	31
Hong Kong	(*26*)	1994–1995	35	28	(7)
	(*27*)	1984–1990	34		(6)
	(*28*)	1983–1984	29		(1)
	(*29*)	1987–1996	26		2
Vietnam	(*30*)	1981–1984	22	59	37

An alternative explanation for the high detection rates could be the strict adherence to standard stool sample collection and handling procedures or the use of more sensitive tests compared to previous studies. Because rotaviruses are relatively stable in whole stool samples and because the studies chosen for comparison of current data all used comparable enzyme-linked immunosorbent assays, we think that the methods used by our network probably had little impact on detection rates. The principal advantage of the use of the standardized surveillance protocols remains the ease with which it allows for initiation of surveillance and enables collection of comparable data from very diverse settings. Finally, the reasons that data from Hong Kong generally reflected lower rates of disease than other sites remain unclear and deserve additional study. These preliminary data highlight the need to collect data in countries considering rotavirus vaccine introduction.

During this study, surveillance for rotavirus was initiated and sustained with ease, even in very large hospitals. The Generic Protocol for Rotavirus Disease Burden Estimation from WHO provides simple guidelines on organizing surveillance and interpreting the data. This protocol formed the basis of this network. Since diagnosis of rotavirus diarrhea is relatively easy compared with other vaccine-preventable diseases, ARSN was able to establish rotavirus testing by using EIA or PAGE at each site. Data collection was simplified by use of a one-page standard data-collection form contained in the Generic Protocol as a template for each site's form and by creation of a premade data entry form and analysis program in EpiInfo (CDC, Atlanta, GA) (available by request from the authors).

The use of regional networks to document rotavirus strain distribution within a region will add to the global understanding of prevalent strains and help in making informed decisions on vaccine composition. Although, strain characterization data from ARSN members during the 12 months of surveillance reported here were not yet complete, knowledge of circulating strains may also help guide local decisions on vaccine introduction and will be important in postlicensure assessment of vaccine effectiveness. Since the leading vaccine candidates employ different strategies, some monovalent human or animal strains and some polyvalent human-animal reassortants, conducting field trials of new vaccines in settings that include diverse, naturally occurring strains will be important. Surveillance networks, such as the ARSN, facilitate data collection for vaccine trials and act as a resource for laboratory scientists.

Regional, cooperative surveillance networks create training and infrastructure, building opportunities for members and creating a mechanism to introduce new technologies. While detecting rotavirus is easy and rapid, characterizing strains requires a higher level of technical skill. ARSN work has been supported by the WHO Collaborating Center for Rotavirus and Other Viral Agents of Gastroenteritis to conduct strain typing for sites without the capacity to do so and to facilitate training and quality control for laboratories interested in performing these tests. When ARSN was formed, four member sites (Thailand National Institute of Health, Chinese University of Hong Kong, National Institute of Hygiene and Epidemiology in Vietnam, and China's Institute of Viral Disease Control and Prevention) had performed rotavirus strain typing. By the end of the first year, strains had been characterized by using RT-PCR by eight of the sites, and scientists from Myanmar were receiving training in this method. In addition, ARSN has provided a platform for professional development for epidemiologists and healthcare personnel involved in the study.

## Future Directions

The final product from ARSN will be data that countries in the region can use to assess their need for a rotavirus vaccine and, where needed, to facilitate their introduction into immunization programs. The work of ARSN can serve as a paradigm of additional regional activities to promote the understanding and appreciation of the disease prevalence and costs associated with rotavirus in the region. Other regional rotavirus surveillance networks are also established in Africa and are being established in Latin America and will help accelerate decisions on vaccine introduction in those regions. The next step is to link these data to advocacy efforts. The value of any surveillance data is in its application or its ability to inform decisions. The new initiative of GAVI, the Accelerated Development and Introduction Program for Rotavirus Vaccines (available from: www.vaccinealliance.org), will serve as a pathway to transform data from networks like ARSN into action.
